# Isolation of Osteoprogenitors from Human Jaw Periosteal Cells: A Comparison of Two Magnetic Separation Methods

**DOI:** 10.1371/journal.pone.0047176

**Published:** 2012-10-19

**Authors:** Marcus Olbrich, Melanie Rieger, Siegmar Reinert, Dorothea Alexander

**Affiliations:** Department of Oral and Maxillofacial Surgery, University Hospital of Tübingen, Tübingen, Germany; Georgia Health Sciences University, United States of America

## Abstract

Human jaw periosteum tissue contains osteoprogenitors that have potential for tissue engineering applications in oral and maxillofacial surgeries. To isolate osteoprogenitor cells from heterogeneous cell populations, we used the specific mesenchymal stem cell antigen-1 (MSCA-1) antibody and compared two magnetic separation methods. We analyzed the obtained MSCA-1^+^ and MSCA-1^−^ fractions in terms of purity, yield of positive/negative cells and proliferative and mineralization potentials. The analysis of cell viability after separation revealed that the EasySep method yielded higher viability rates, whereas the flow cytometry results showed a higher purity for the MACS-separated cell fractions. The mineralization capacity of the osteogenic induced MSCA-1^+^ cells compared with the MSCA-1^−^ controls using MACS was 5-fold higher, whereas the same comparison after EasySep showed no significant differences between both fractions. By analyzing cell proliferation, we detected a significant difference between the proliferative potential of the osteogenic cells versus untreated cells after the MACS and EasySep separations. The differentiated cells after MACS separation adjusted their proliferative capacity, whereas the EasySep-separated cells failed to do so. The protein expression analysis showed small differences between the two separation methods. Our findings suggest that MACS is a more suitable separation method to isolate osteoprogenitors from the entire jaw periosteal cell population.

## Introduction

Bone grafts used in alveolar and jawbone reconstruction can be generated through tissue engineering approaches [1]. Osteoprogenitor cells may serve as a suitable cell source for this purpose. The periosteum contains multipotent mesenchymal stem cells (MSCs) that show the potential to differentiate into different lineages, such as adipogenic, chondrogenic, myogenic and osteogenic tissues [2].

The periosteum consists of three zones. The osteogenic progenitors are restricted to the inner layer, known as the cambium layer, whereas the following zone mainly contains tissue fibroblasts. The third zone consists primarily of collagen fibers [3], [4]. Although a reliable separation of the different layers is difficult due to the low thickness of human jaw periosteum tissue, identifying characteristic markers for the osteogenic progenitors is important to ensure the success of future clinical applications using this stem cell type.

We recently showed that MSCA-1^+^ (mesenchymal stem cell antigen-1)-enriched human jaw periosteum-derived cells (JPCs) had a higher osteogenic potential compared with MSCA-1^−^ cells [5]. MSCA-1 is expressed by the mesenchymal stem cells of the CD271^bright^ subset population within bone marrow [6], [7]. MSCA-1 was identified as the tissue non-specific alkaline phosphatase (TNAP) [8].

Previous work by Mucci et al. [9] compared magnetic separation methods. In their report, isolated CD14^+^ monocytes from blood were used to generate *in vitro* dendritic cells (DC), and the MACS and EasySep isolation systems were compared. Despite the high purity and the same viability of both methods, the authors recommended MACS as being more suitable for obtaining non-activated DCs for functional studies.

In the present study, we compared two magnetic separation methods for obtaining a pure osteoprogenitor subpopulation using the MSCA-1 specific antibody. Due to the relatively low survival rates obtained by MACS (Miltenyi Biotec, Bergisch Gladbach, Germany), we compared it with the EasySep approach provided by Stem Cell Technologies (Vancouver, Canada) and analyzed survival rates, cell purities, yields, *in vitro* mineralization potential and proliferative capacities of isolated fractions.

## Materials and Methods

### Cell culture

Human jaw periosteum was obtained during routine maxillofacial biopsies after written informed consent. Samples from 16 donors were included in this study in accordance to the local ethical committee (Ethik-Kommission der Medizinischen Fakultät Tübingen, approval number 194/2008BO2). After the main digestion step using type XI collagenase (1500 U/ml, Sigma, Steinheim, Germany) for 90 min, the JPCs were plated into 75 cm^2^ culture flasks. Until reaching confluence, the JPCs were cultured in DMEM/F-12 (Invitrogen-BioSource Europe, Nivelles, Belgium) containing 10% FCS (Sigma-Aldrich, Steinheim, Germany), 1% fungicide and penicillin/streptomycin (Biochrom, Berlin, Germany). The cells were passaged using trypsin-versene EDTA (1×, Lonza, Basel, Schweiz). The JPCs from the fifth to seventh passages were used in the subsequent experiments. Only JPCs that were able to mineralize *in vitro* were included in this study.

### Differentiation experiments

For the osteogenic differentiation experiments, the JPCs (3.5×10^4^ cells per well in 6-well plates) were cultured in OB medium (DMEM/F12 containing 10% FCS, 10 mM β-glycerophosphate, 100 µM L-ascorbic acid 2-phosphate and 4 µm dexamethasone (Sigma-Aldrich, Steinheim, Germany)). The cells were treated with these supplements for 30 days. The medium was replaced three times per week. Untreated cells, cultivated without any osteogenic compounds for the same period, served as undifferentiated controls.

### Magnetic cell labeling and separation of the MSCA-1^+^ cell fraction by MACS and EasySep

For separation of the MSCA-1^+^ cell fraction from the entire JPC population, two magnetic separation approaches were compared. The JPCs were labeled using either the anti-MSCA-1 MicroBead Kit (Miltenyi Biotec, Bergisch Gladbach, Germany) or the human “do-it-yourself” selection kit (StemCell Technologies, Cologne, Germany) in combination with the anti-MSCA-1 antibodies (Miltenyi Biotec, Bergisch Gladbach, Germany).

For the MACS separation, up to 2×10^8^ total cells were centrifuged at 300×*g* for 10 min. The cell pellets were resuspended in 1200 µl of phosphate-buffered saline (PBS, pH 7.2), 0.5% bovine serum albumin (BSA) and 2 mM EDTA. FcR blocking reagent (400 µl) and anti-MSCA-1 MicroBeads (400 µl) were added, and the mixture incubated for 20 min at 4°C. After washing with 1 ml of PBS buffer, the cell suspensions were centrifuged at 300×*g* for 10 min. The labeled cells were resuspended in 1 ml of PBS buffer. The samples were first applied to pre-separation filters to remove cell clumps and then onto MS columns. The unlabeled MSCA-1^−^ cells passed through the column, whereas the MSCA-1^+^ cells remained in the column due to the magnetic field. After triple washing with 500 µl of PBS buffer, the column was removed from the magnetic field, and the MSCA-1^+^ fraction was eluted.

For the EasySep separations, up to 1×10^8^ total cells were incubated for 15 min with 100 µl of a positive selection cocktail containing anti-MSCA-1 antibodies. The EasySep magnetic nanoparticles (55 µl) were added to the cell-antibody mixture, and it was incubated for 10 min at room temperature. Buffer (PBS, 2% FCS, 1 mM EDTA) was added to the cell suspension to reach a volume of 2.5 ml. The samples were placed into the magnetized chamber, and the samples incubated for 10 min. The magnet was inverted to collect the MSCA-1^−^ fraction, and the MSCA-1^+^ cell fraction remained inside the tube. This EasySep procedure (adding 2.5 ml buffer, incubating with the magnet and inverting the magnet) was repeated three times. The tube was then removed from the magnet, and the remaining cells were resuspended in the culture medium. Following both separation methods, 3.5×10^4^ cells of each fraction were plated per well in a 6-well plate for further experiments.

### Flow cytometry analysis of MSCA-1 expression in the MACS- and EasySep-separated cell fractions

The separated cell fractions (MSCA-1^+/−^) were centrifuged (350×*g*, 7 min), resuspended in 20 µl of 10% Gamunex (human immune globulin solution, Talerics Biotherapeutics, Frankfurt, Germany) and incubated for 15 min at 4°C. After adding 100 µl of FACS buffer (PBS, 0.1% BSA, 0.1% sodium azide), the cells were mixed with anti-MSCA-1 supernatants [10] and incubated for 15 min at 4°C. The cells were centrifuged (350×*g*, 7 min) and washed two times with FACS buffer. The cell pellets were then incubated with 10 µl of the secondary phycoerythrin (PE)-labeled polyclonal goat anti-mouse antibodies (Dako, Glostrup, Denmark) for 15 min at 4°C. The cells were washed two times, centrifuged and resuspended in 200 µl of FACS buffer for flow cytometry analysis. The measurements were carried out with a FACScan (Becton Dickinson, Franklin Lakes, USA). The negative control cells were only incubated with the secondary antibodies. For data evaluation, CellQuestPro software was used.

### Detection of the mineralization capacity for the MACS- and EasySep-separated cell fractions

Alizarin Red staining was used for the detection of calcium phosphate precipitates in the MACS and EasySep separated cell fractions. The cells were fixed in methanol (−20°C) for 2 min and air-dried. They were then incubated with the Alizarin Red dye (40 mM, pH 4.0) for 20 min. After washing with deionized water (four times for 5 min on a shaker), the cells were rinsed in PBS, dehydrated with ethanol (−20°C) and air-dried. The calcium phosphate precipitates were stained bright red.

For quantification of Alizarin Red staining, a commercial osteogenesis quantification kit (Millipore, Billerica, U.S.A.) was used according to the manufacturer's instructions. Briefly, Alizarin Red dye was dissolved from the monolayer by adding 10% acetic acid to each well and shaking for 30 min. Cell monolayers were detached using cell scrapers, and the cell solutions were transferred to microcentrifuge tubes. The samples were heated for 10 min at 85°C and then cooled on ice for 5 min. After centrifuging (20,000×*g*, 15 min), the supernatants were transferred to fresh tubes and neutralized with 10% ammonium hydroxide. To quantify the calcium phosphate deposits, optical density measurements were acquired at a wavelength of 405 nm for a dilution series of known concentrations (30 µM, 15 µM, 7.5 µM, 3.75 µM, 1.88 µM, 0.94 µM, 0.47 µM) of the Alizarin dye using an ELx800 ELISA Reader (Bio-Tek, Winooski, U.S.A.). KC4 software was used for data evaluation.

### Analysis of cell proliferation in MACS- and EasySep-separated cell fractions

A commercial available kit (EZ4U, Biomedica, Vienna, Austria) was used to determine the cell proliferation rates for the MACS and EasySep separated cell fractions (MSCA-1^+/−^) during osteogenesis with the undifferentiated controls. After the cell separations, 1.5×10^3^ cells were seeded per well in 96-well plate in triplicates for each obtained fraction. After an overnight incubation for cell adhesion, the culture medium was removed, and medium with or without osteogenic stimuli (OB) was added. After the different incubation times (day 5, 10 and 20) substrate (20 µl) was added to each well, and the mixture was incubated for 4 h at 37°C. For the quantification of cell proliferation, optical density measurements at a wavelength of 450 nm and at wavelength of 620 nm as a reference were carried out using an ELx800 Elisa Reader (Bio-Tek, Winooski, U.S.A.). KC4 software was used for data evaluation.

### Protein expression analysis in MACS- and EasySep-separated cell fractions

For the parallel detection of different soluble receptors and related proteins, a commercial proteome profiler array (R&D Systems, Abingdon, U.K.) was used. The nitrocellulose membranes were incubated overnight at 4°C on a rocking platform shaker with the supernatants from the MACS and EasySep separated cell fractions. The membranes were washed three times for 10 min using the provided wash buffer and were incubated with the detection antibody cocktail for 2 h. After a repeated washing, the membranes were incubated with streptavidin-horse radish peroxidase (HRP) for 30 min and were then exposed to chemiluminescent reagents (ECL Plus Western Blotting Detection System, GE Healthcare; Munich, Germany) for 5 min and to X-ray films for 60, 90 and 120 seconds. The pixel intensities of the positive signals were analyzed using ImageJ 1.4 software and the plugin DotBlotAnalyzer.

### Statistical analysis

The data are expressed as the mean ± SEM. For the statistical analysis, Student's t-test and Welch's approximate t were used. A p-value<0.05 was considered significant.

## Results

### Differentiation potential of analyzed cell culture specimens

For the explicit comparison of MACS and EasySep cell separation methods only JPCs that were able to mineralize *in vitro* were included in this study. The osteogenic differentiation potential of the JPCs was determined by Alizarin and von Kossa stainings (after 20 days of osteogenic induction) and the expression of alkaline phosphatase, as published previously [5], [10].

### Determination of the survival rates after MACS and EasySep cell separations

**Figure 1:**
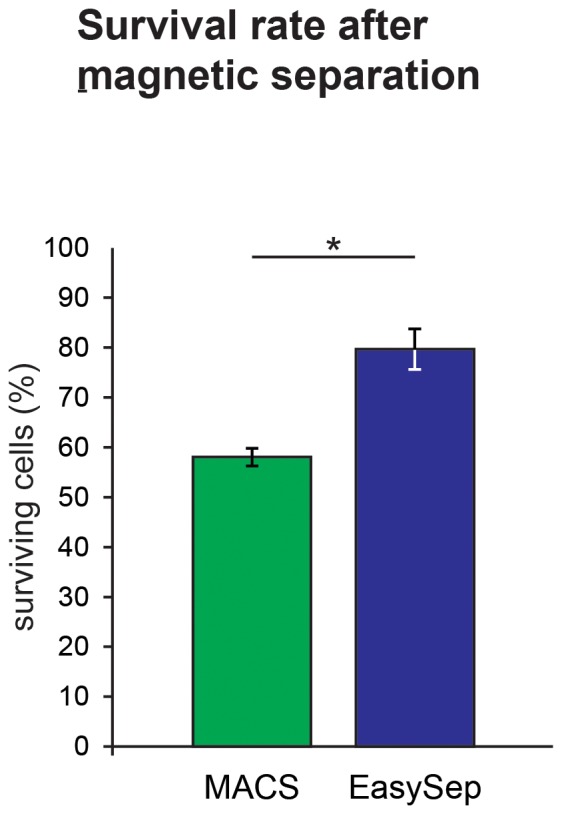
Survival rates after MACS (green column) and EasySep (blue column) separation. For the MACS separation, about 40% and for the EasySep separation about 20% of the initially applied cells were lost. The difference between the survival/mortality rates obtained by both separation methods (n = 16 for each separation method) was significant (p<0.0001).

The cell counts of the JPCs isolated from the donors (n = 16) were determined before each MACS and EasySep magnetic separation and directly afterwards. The survival and mortality rates were calculated for each separation method. For the MACS separation, 58,02%±1,75 of the cells survived, giving a mortality rate of approximately 40% as shown in Fig. 1. For the EasySep separation, 79,68%±4,06 of the cells survived, indicating that about 20% of the initially applied cells was lost. The difference between the survival/mortality rates obtained by both separation methods was significant (p<0.0001).

### Analysis of cell purity of MACS- and EasySep-separated cell fractions

**Figure 2:**
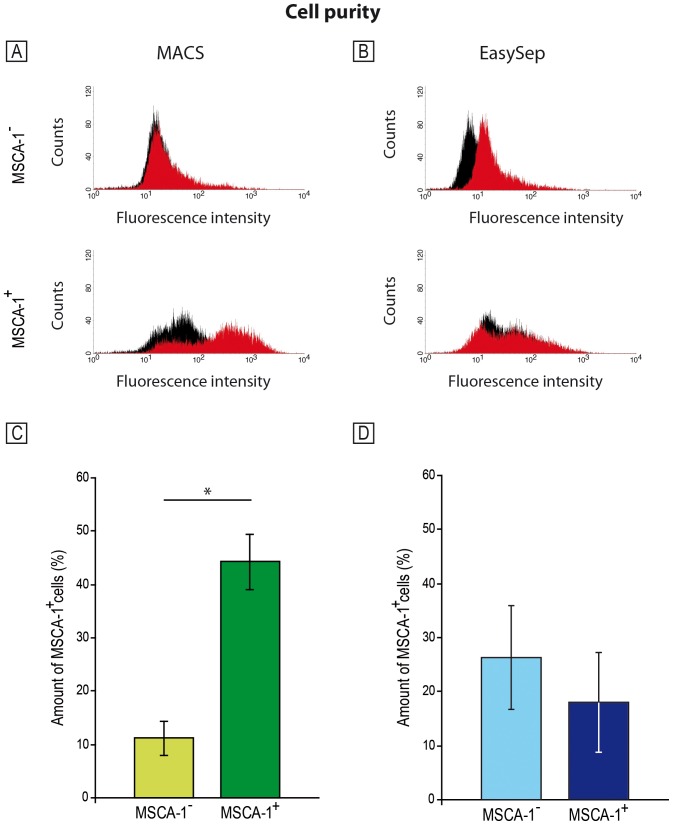
Cell purity of MACS (A, C) and EasySep (B, D) separated cell fractions as determined by flow cytometric analysis. After the magnetic separations, the cells were labeled with the anti-MSCA-1 supernatants and the PE-labeled secondary antibodies and MSCA-1 expression was analyzed by FACS. In the upper panel of the figure, representative flow cytometric histograms of MSCA-1^−/+^ are illustrated. Fig. 2C and 2D show the results of the quantification of the MSCA-1^+^ cells within the MACS/EasySep separated fractions (n = 4). MSCA-1 expression was significantly different (p<0,021) between the MACS- but not EasySep-separated MSCA-1^−^ (11.15±3.17) and MSCA-1^+^ (44.26±5.24) cell fractions.

The purities of the separated MSCA-1^−^ and MSCA-1^+^ cell fractions were analyzed using flow cytometry analysis. After the magnetic separations, the cells were labeled again with the anti-MSCA-1 supernatants and the PE-labeled secondary antibodies. MSCA-1 expression was analyzed using flow cytometry. Figs. 2A–B show representative histograms of the MACS (Fig. 2A) and EasySep (Fig. 2B) separated cell fractions (black – PE control, red – MSCA-1 stained sample). The MSCA-1^−^ cells separated by MACS show a very weak MSCA-1 expression, whereas a clear shift in MSCA-1 expression was detected in the positive fraction (Fig. 2A). In contrast, the EasySep separated MSCA-1^−^ cells were shown to partially express MSCA-1, while the positive fraction failed to express the surface marker (Fig. 2B). Fig. 2C and 2D show the results of the quantification of the MSCA-1^+^ cells within the MACS/EasySep separated fractions (n = 4). The y-axis denotes the percentage of MSCA-1 positive cells (%). MSCA-1 expression was significantly different (p<0,021) between the MSCA-1^−^ (11.15±3.17) and MSCA-1^+^ (44.26±5.24) cell fractions after the MACS separation (p<0.0016). We were not able to detect a distinctly higher MSCA-1 expression in the MSCA-1^+^ (18,95±9,73 versus MSCA-1^−^: 27,69±10,10; n.s.) cell fraction after the EasySep separation.

### Quantification of the mineralization capacity of the MACS- and EasySep- separated cell fractions

**Figure 3:**
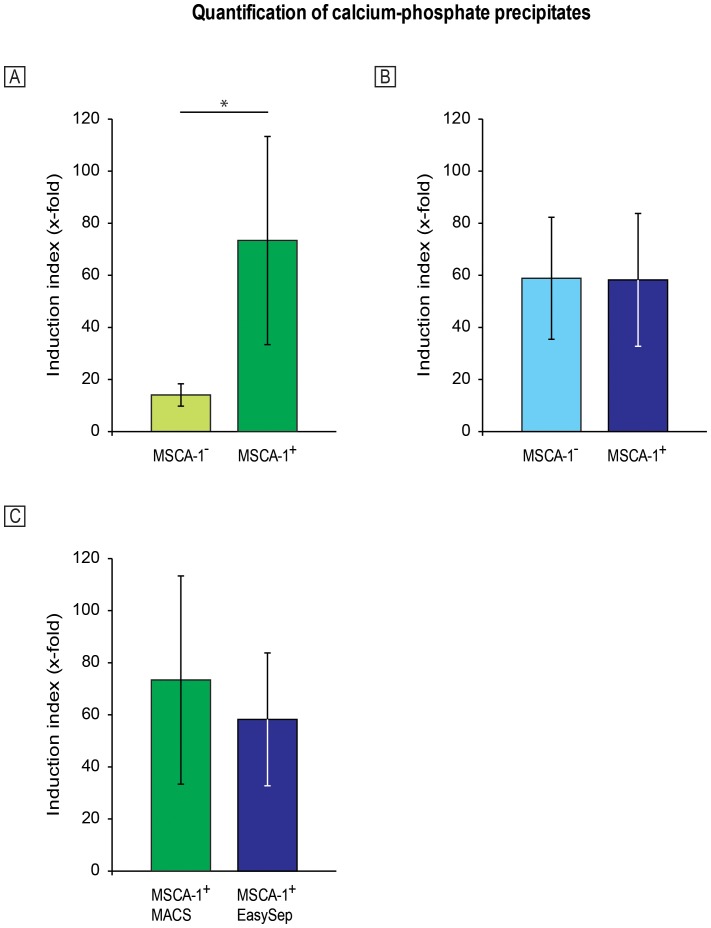
Quantification of the mineralization capacity of the MACS- (bright and dark green columns) and EasySep- (bright and dark blue columns) separated cell fractions. After Alizarin Red staining and subsequent dissolving of the dye from the monolayers, calcium phosphate precipitates were photometrically quantitated (n = 13 for each separation method). The increase in amounts of calcium-phosphate precipitates compared with the untreated controls (induction index, x-fold) is illustrated. A 5-fold higher amount of precipitates was detected in the MACS-separated MSCA-1^+^ compared with the MSCA-1^−^ cells (p<0.05) (A). In contrast, EasySep-separated cell fractions did not significantly differ concerning their mineralization capacity (B). The direct comparison of the positive fractions separated by both methods showed no significant differences, as shown in part C of the figure.

After separation of the MSCA-1^+/−^ cell fractions using both separation methods, the cells were seeded onto 6-well plates and underwent osteogenic differentiation for 30 days. After Alizarin Red staining of the monolayers, calcium phosphate precipitates were photometrically quantitated (n = 13 for each separation method). In general, the MSCA-1^+^ fractions obtained by both separation methods showed enhanced amounts of calcium phosphate precipitates compared with the respective untreated controls, as expected. As shown in Fig. 3, the MACS-separated MSCA-1^−^ cell fractions showed an increase in calcium phosphate precipitates compared with the untreated controls (induction index 14,05±4,29-fold), whereas the MACS-separated MSCA-1^+^ cells revealed 73,37±39,98-fold increase in the number of nodules compared with the undifferentiated controls. Surprisingly, after separation by EasySep, the MSCA-1^−^ cells had a 58,86±23,41-fold increase in the amount of precipitates compared with the untreated controls, whereas an 58,24±25,52-fold increase in nodules was obtained in the MSCA-1^+^ cells. These results indicate that the MSCA-1^+/−^ cell fractions separated by EasySep showed nearly the same mineralization capacity (Fig. 3B). Compared to the results obtained by EasySep, a distinct difference was found between the mineralization capacity of the MSCA-1^+^ and MSCA-1^−^ cell fractions after the MACS separation: we detected a 5-fold higher amount of precipitates in the MACS-separated MSCA-1^+^ compared with the MSCA-1^−^ cells (Fig. 3A, p<0.05). However, the direct comparison of MACS- and EasySep separated MSCA-1^+^ cells concerning the osteogenic capacity revealed no significant differences, as shown in Fig. 3C.

### Analysis of cell proliferation in MACS- and EasySep-separated cell fractions

**Figure 4:**
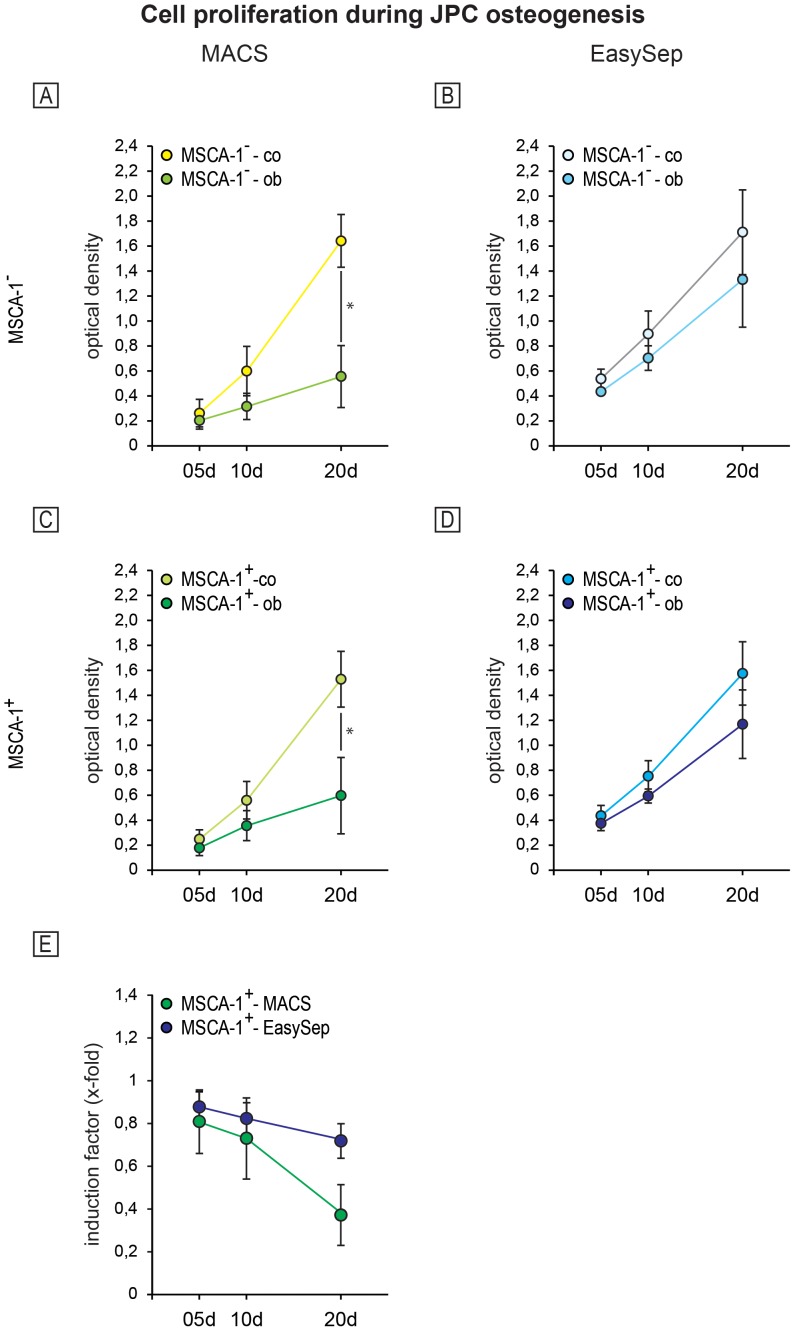
Cell proliferation behaviour during JPC osteogenesis. The proliferation rates (optical densities) of the MACS- (A, C) and EasySep-separated (B, D) MSCA-1^+/−^ cells were analyzed at days 5, 10 and 20 of the osteogenic differentiation (n = 4 for each separation method) compared with the untreated controls. The MACS-separated MSCA-1^−/+^ cells at day 20 showed significant differences (p<0,044) between the undifferentiated cells and the osteogenic induced ones for the MSCA-1^−^ and MSCA-1^+^ cells. In contrast, the EasySep-separated MSCA-1^−/+^ cells proliferated almost identically in osteogenic stimulated and untreated controls. The significant decrease in proliferation of the MACS-separated compared to the EasySep-separated positive fraction at day 20 is shown in part E of the figure (p<0.05).

The proliferation rates of the MACS- and EasySep-separated MSCA-1^+/−^ cells were analyzed at days 5, 10 and 20 of the osteogenic differentiation (n = 4 for each separation method) compared with the untreated controls. The MACS-separated cells showed similar proliferation behavior at day 5 (MSCA-1^−^ co versus OB: 0,26±0,11 versus 0,20±0,07; MSCA-1^+^ co versus OB: 0,24±0,08 versus 0,18±0,06, Fig. 4A and 4C). The distinct differences between the untreated and osteogenic induced cells were detected at days 10 (MSCA-1^−^ co versus OB: 0,60±0,20 versus 0,32±0,11; MSCA-1^+^ co versus OB: 0,56±0,15 versus 0,36±0,126) and 20 (MSCA-1^−^ co versus OB: 1,65±0,33 versus 0,56±0,25; MSCA-1^+^ co versus OB: 1,53±0,22 versus 0,60±0,31). In general, the undifferentiated cells proliferated faster than the osteogenic induced ones. With the exception of day 20 of osteogenesis, no significant differences were detected between the proliferation rates from the MSCA-1^+^ and the MSCA-1^−^ cell fractions at day 5 and 10. The MACS-separated cells at day 20 showed significant differences (MSCA-1^−^: p<0,044; MSCA-1^+^: p<0,044) between the undifferentiated cells and the osteogenic induced ones for the MSCA-1^−^ and MSCA-1^+^ cells.

The EasySep-separated cell fractions have similar proliferations (nearly parallel) as illustrated by the curve progressions in Fig. 4B and 4D at days 5 and 10 (MSCA-1^−^ co versus OB: 0,90±0,18 versus 0,70±0,10; MSCA-1^+^ co versus OB: 0,75±0,13 versus 0,60±0,06). Minor differences were detected at day 20 (MSCA-1^−^ co versus OB: 1,72±0,34 versus 1,34±0,38; MSCA-1^+^ co versus OB: 1,58±0,25 versus 1,17±0,28). However, no significant differences in the proliferation rates were found between the undifferentiated and osteogenic induced cells or between the MSCA-1^+^ and the respective MSCA-1^−^ cell fraction. The comparison of the positive fractions separated by both methods revealed a clear difference of the decrease in proliferation for the MACS-separated fraction at day 20 however, the difference was not significant as shown in Fig. 4E.

### Protein expression analysis

**Figure 5:**
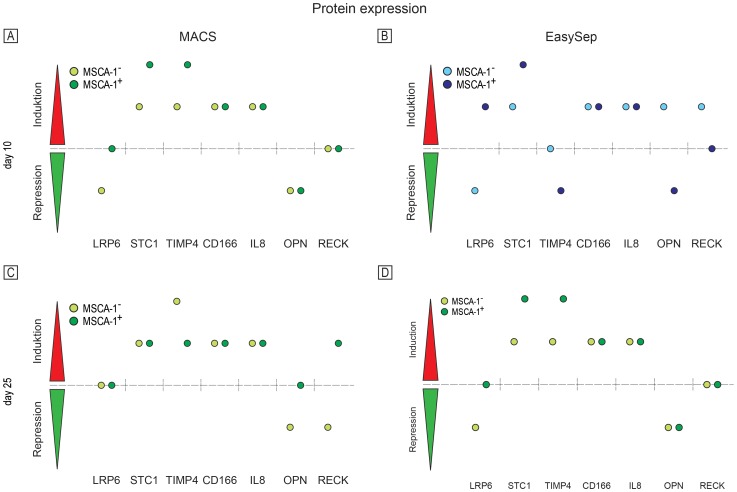
Protein expression analysis in supernatants from MACS- (A, C, bright and dark green spots) and EasySep-separated (B, D, bright and dark blue spots) MSCA-1^+/−^ cells were analyzed at days 10 and 25 of the osteogenic differentiation (n = 3 for each separation method) compared with the untreated controls. Protein expressions of LRP-6, STC-1, TIMP-4, CD166, IL-8, OPN and RECK were illustrated over the baseline in the case of induction (red) and under the line in the case of repression (green).

The supernatants of the differentiating and magnetically separated JPCs after days 10 and 25 of osteogenesis were used for the analysis of different soluble receptors and related proteins. The osteogenic induced cells were compared with the corresponding untreated controls for the analysis of receptor or protein expression (Fig. 5).

At day 10, the expression of low-density lipoprotein receptor-related protein 6 (LRP-6) in the MACS-separated MSCA-1^+^ cells showed no difference (OB-treated versus untreated), whereas it was downregulated in the MSCA-1^−^ fraction. Stanniocalcin 1 (STC-1) and the tissue inhibitor of metalloproteinase-4 (TIMP-4) were induced in the MSCA-1^−^ cells, and it was shown to be upregulated in the MSCA-1^+^ cell fraction. Activated leukocyte cell adhesion molecule (ALCAM/CD166) and interleukin-8 (CXCL8/IL-8) showed a relatively high protein release in the MSCA-1^−/+^ fractions. Osteopontin (OPN) was repressed in the positive and negative fractions, whereas the expression of reversion-inducing cysteine-rich protein with Kazal motifs (RECK) showed no differences compared with the untreated controls.

The expression of LRP-6 was unchanged in the MACS-separated MSCA-1^−/+^, but STC-1 expression showed still elevated protein levels in both fractions after day 25 of osteogenesis. However, TIMP4 expression remained upregulated in both fractions. Higher levels were detected in the MSCA-1^−^ fraction. We found no alterations in CD166 and IL-8 expression compared with those after day 10 of osteogenesis. The OPN levels were repressed in the MSCA-1^−^ cells, and the positive fraction showed no differences compared with the untreated controls. RECK expression was repressed in the MSCA-1^−^ cells, and it was induced in the MSCA-1^+^ cells.

After EasySep separation, LRP-6 showed at day 10 of osteogenesis a repressed expression pattern in the MSCA-1^−^ fraction, but it was shown to be upregulated in the MSCA-1^+^ cells. The expression profile of STC-1 was unchanged after MACS-separation. Both fractions were induced, but MSCA-1^+^ showed higher expression. For TIMP-4, no differences were detected in the MSCA-1^−^ cells, whereas TIMP-4 expression was downregulated in the MSCA-1^+^ cells. CD166 and IL-8 showed a similar expression pattern after MACS separation (10 days), we detected upregulated levels in both fractions. OPN was induced in MSCA-1^−^, but it was repressed in the MSCA-1^+^ cells. RECK showed no differences in the MSCA1^+^ cells, but it did show an upregulation in the MSCA-1^−^ cell fraction.

LRP-6 showed the same expression regulation at day 25 compared with day 10 of osteogenesis. STC-1 was upregulated to a similar level in both fractions. TIMP-4 showed no differences in the MSCA-1^−^ cells but was induced in the MSCA-1^+^ cells. Similarly, upregulated levels were detected for CD166 and IL-8 in both fractions with higher levels in the MSCA-1^+^ fraction. The MSCA-1^+^ cell fraction showed increased levels of OPN and RECK expression.

## Discussion

To ensure the quality of prospective clinical tissue engineering applications a detailed characterization of stem cells to be transplanted is essential. We have previously shown [5] that the MSCA-1^+^ cell fraction exhibits a higher osteogenic capacity compared with the MSCA-1^−^ subpopulation within the heterogeneous population of JPCs. The MSCA-1^+^ cell fraction represents a rare subpopulation, and the obtained cell yields after MACS separation are low with high mortality rates. In the present study, we compared two magnetic separation methods, MACS and EasySep, for their ability to obtain a pure osteoblast progenitor cell population from JPCs. Using the EasySep separation, we obtained higher cell survival rates, whereas MACS provided higher purity for the isolated subpopulations. The quantification of the calcium phosphate precipitates showed significant higher amounts of calcium precipitates in the MACS-separated MSCA-1^+^ fraction compared with the MSCA-1^−^ fraction. In contrast, we detected no significant difference concerning the osteogenic potential of EasySep-separated cell fractions. The analysis of the proliferative capacity of the MACS-isolated subpopulations showed a clear decrease in the proliferation after the induction of osteogenesis, whereas the EasySep subpopulations proliferated almost identically, irrespective of the culture treatment. The EasySep-separated fractions did not alter their proliferative potential during osteogenesis; therefore, we conclude that they pass through an incomplete differentiation process. The protein expression data show no conclusive pattern and are difficult to interpret due to the poor purity of EasySep fractions. Our findings suggest that magnetic separation by the standard for cell separations MACS provides a purer MSCA-1^+^ cell fraction that can differentiate into the osteogenic lineage.

The detected differences in the surviving cells were not donor-dependent because the cells of one donor were divided immediately before the two separation methods were performed. We compared both methods on the basis of the same number of cells cultured and treated identically. The early experiments revealed a dependency between the separation methods and survival rates rather than a correlation between the donor and the separation method.

Alipoor and colleagues [11] used the MACS technology to isolate spermatogonial stem cells from prepubertal mouse testis. They obtained survival rates of 92.52%. Mucci et al. [9] measured high viability rates for the positive selected human CD14^+^ monocytes using EasySep and MACS. However, the dextran-coated beads associated with the EasySep product probably induced an early stage of dendritic cell maturation in the absence of a maturation stimulus. Both separation methods provided pure and viable dendritic cells but the authors recommended the MACS system for functional assays. Kim et al. [12] separated murine CD45^+^ cells from bone marrow using a droplet-based magnetically activated cell separator (DMACS) and MACS. After magnetic separation, they calculated the number of dead cells. Using DMACS, 94.59% of the cells survived, whereas by MACS only 62.69% of the cells survived. We detected 80% surviving cells after EasySep, whereas our MACS results are similar to those published by Kim et al. (60% surviving cells). Our JPCs may be more likely to be damaged during the MACS separation. The lower survival rates of the separated JPCs by MACS probably originates from the shearing forces occurring while the cells flush through a small opening in the column, whereas the cell suspension during separation by EasySep rests in a buffer-filled tube with a larger opening. These cells experience less shearing force.

Pafumi et al. [13] published their data on the enrichment of CD34^+^ stem cells from umbilical cord blood. Two column-based separation methods were used: Stem Sep (StemCell Technologies, Cologne, Germany) for negative selection and Mini MACS for positive cell selection. The latter achieved a purification efficiency of 99%, and Stem Sep isolation had a purification of only 80%. Handgretinger et al. [14] used MACS to isolate CD133^+^ cells from peripheral blood, and they obtained a purity of 94%. Mucci et al. [9] reported cell purities higher than 90% for CD14^+^ cells in the enriched fraction by MACS and EasySep. We determined the amount of positive cells within the MSCA-1^+^ cell fraction after the MACS and EasySep separation by FACS analysis and obtained averages of 44% and 11%, respectively. The low purity rates are probably due to the adhesivity of the JPCs, which prevents a clear separation of the positive and negative cells. The MSCA-1 antibody may recognize an antigen that is expressed on all of the JPCs at different level, which could hamper clear separation. For the differences in purity between the MACS and EasySep methods, the column setup likely provides higher cell purity because the buffer runs through the column to wash off and extract any magnetically unlabeled cells. EasySep offers the possibility for unlabeled cells to be easily removed from magnetically labeled cells. However, the three inversions of the magnet within 30 minutes as recommended by the manufacturer may not be sufficient to collect all of the unlabeled cells. As shown in Fig. 2, the magnetically labeled cells apparently merge into the negative cell fraction (28% of the cells from the MSCA^−^ fraction are positive). Reciprocally, we found that 19% of the MSCA^+^ fraction is positive. The clearly lower purification efficiency using EasySep could be due to a lower magnetic field in the interior of the cavity or to weaker magnetic beads for the labeling of the samples.

After the quantification of the mineralization capacity, we found a 5-fold higher osteogenic capacity of MSCA-1^+^ compared with the MSCA-1^−^ cells after MACS separation, whereas EasySep separated cells showed no significant difference between both fractions. However, we detected a delayed mineralization process compared to that from unsorted cells after MACS separation. One reason for this observation might be the continued recovery after the higher shear stress and the subsequent higher number of non-viable cells after MACS separation. Although the MSCA-1^+/−^ cells were seeded with the same density before the induction of osteogenesis, we observed a lower cell adherence of MSCA-1^+^ compared with the MSCA-1^−^ cell fraction at the beginning of differentiation, indicating that the cell viability of this fraction was decreased relative to the negative fraction (unpublished data). Due to this observation, the entire differentiation process might be delayed, thus explaining the delayed mineralization process.

In terms of proliferation, only the MACS fraction showed a significant difference regarding the proliferation behavior of the osteogenic induced cells compared with the untreated controls (for the MSCA-1^−^ and MSCA-1^+^ cell fractions). In contrast, the EasySep separated osteogenic induced cells proliferated almost identically to the untreated control cells. Cell proliferation potential decreases with increasing differentiation. The MACS method seems to maintain the differentiation potential of the cells, whereas the EasySep method could not maintain this property. On the other site, we expected differences concerning the proliferation potential of the MSCA-1^+^ and MSCA-1^−^ cell fraction, but because we did not detect any differences, we concluded that the positive and negative fraction contain osteogenic progenitors that display the capacity to differentiate. These results were confirmed by the Alizarin Red staining, which showed weaker but not completely negative staining of the MSCA-1^−^ cell fraction.

The interpretation of our protein expression results is speculative because we cannot exactly define the magnetically obtained subpopulations by the EasySep method. Therefore, we focus this discussion part only on MACS-separated cells. The low-density lipoprotein receptor-related protein 6 (LRP-6) functions as a co-receptor for Wnt signal transduction and is considered to be a component of the Wnt receptor complex [15], [16]. The canonical Wnt signal pathway also regulates the differentiation of mesenchymal precursors via osteochondro-progenitors into osteoprogenitors [17], [18]. Mice with a heterozygous mutation of LRP-6 showed reduced bone accrual and bone mass compared with WT mice [19]. The MACS-separated MSCA-1^+^ fraction showed higher expression levels of LRP-6 compared with the MSCA-1^−^ cells at day 10, on day 25 expressions were the same. The analysis of LRP-6 expression in the MACS-separated fractions indicates that this receptor-related protein initiates the osteogenic process and afterwards declines in expression at day 25.

In 1996, Olsen et al. [20] discovered an evolutionary conserved trait between fish and mammalian stanniocalcin, suggesting that mammalian stanniocalcin was a regulator of mineral homeostasis. Yoshiko et al. showed that stanniocalcin 1 (STC-1) is expressed at higher levels in cells involved in the osteogenesis of mouse embryos and, therefore, suggested that STC-1 is involved in endochondral and intramembranous bone formation [21]. These data support our results showing an upregulation in all osteogenic induced cell fractions, regardless of the separation method. The MSCA-1^+^ cells had higher STC-1 levels compared with the negative control group at day 10 of osteogenesis, whereas STC-1 expression remained upregulated compared with the untreated controls but did not differ in both fractions at day 25.

Tissue inhibitors of metalloproteinases (TIMPs) are the key inhibitors of matrix metalloproteinases (MMPs) in tissues and play a role in the remodeling of the extracellular matrix [22]. TIMP4 functions in a tissue-specific manner as part of a response to tissue remodeling and inhibits MMP-2 and MMP-7 and less intensively for MMP-1, MMP-3 and MMP-9 [23]. Our recent findings suggest an involvement of TIMP4 in ECM remodeling during JPC osteogenesis [24]. Comparing these findings with the results presented here, a strong upregulation of TIMP4 was detected after MACS separation at day 10 as shown by our earlier results. At day 25 of JPC osteogenesis, TIMP4 was still upregulated; however, the expression pattern was reversed. The MSCA-1^−^ fraction showed higher TIMP4 levels compared with the positive fraction.

In 1995, Bowen and colleagues reported the characterization of activated leukocyte cell adhesion molecule (ALCAM/CD166) and found that it was expressed by activated leukocytes. Battula et al. [7] described the expression of CD166 on MSCA-1^+^CD56^+/−^ human mesenchymal stem cells isolated from bone marrow. Choi et al. isolated periosteum-derived progenitors that were triple positive for CD9, CD90 and CD166 [25]. Their results suggest that CD9, CD105 and CD166 can be used to isolate progenitors from periosteum-derived cells as an alternative stem cell source for allograft cellular therapy. Dennis et al. correlated the expression of cell surface molecules, such as CD166, CD105 and CD90, on human bone marrow-derived stem and progenitor cells with increased *in vivo* (SCID mice) bone formation scores after implantation on a ceramic matrix material [26]. We found a higher expression of CD166 in all of the osteogenic induced cell fractions compared with the control cells, regardless of the separation method.

In addition to being a mediator of the inflammatory response, interleukin-8 (CXCL8/IL8) functions as a potent promoter of angiogenesis [27], [28]. Working with human cultured periosteal sheets, Kawase et al. found an upregulation of IL8 during osteogenic differentiation [29]. An increasing number of blood vessels lead to more mature osteoblasts and, therefore, to increased bone formation [30]. As a result of osteogenic stimulation, the MSCA-1^−/+^ cell fractions after the MACS separations showed an upregulation of IL8 compared with the untreated controls.

Chen et al. compared human metaphyseal periosteum-derived cells with bone marrow-derived stem cells from the same donor [31]. After osteogenic induction *in vitro*, they detected more robust mineralization and higher mRNA levels of BMP2, osteopontin (OPN) and osteocalcin in the periosteum-derived cells compared with the bone marrow-derived cells. OPN binds cell surface integrin receptors and is involved in the regulation of mineralization [32]. Osteopontin is increased as osteoblasts develop. Liu et al. also found moderate to high expression levels of OPN in early osteoprogenitors and preosteoblasts [33]. We detected higher OPN expression at day 25 in the MSCA-1^+^ fraction after MACS separation compared with day 10 and with the MSCA-1^−^ fraction.

Reversion-inducing cysteine-rich proteins with Kazal motifs (RECK) are membrane-anchored glycoproteins that function as a negative regulator for MMP-9. RECK also inhibits MT1-MMP and MMP-2 [34]. Similar to TIMP4, it is involved in ECM remodeling during osteogenesis [29]. Oh et al. showed that in a human fibrosarcoma-derived cell line (HT1080), high RECK and low MMP activity occurs in areas where angiogenesis is suppressed and that lower RECK and higher MMP activity are predominant in areas where angiogenesis normally occurs [34]. We found the highest RECK expression levels in the MSCA-1^+^ fractions at day 25 after MACS separation. We cannot state that angiogenesis is suppressed at this time point, but ECM remodeling is occurring. Taken together, we detected higher LRP-6 levels and a stronger induction of STC-1 and TIMP-4 protein levels in the MACS-separated positive fraction compared to the negative one at the beginning of the osteogenesis (day 10), whereas higher OPN and RECK levels were detected to a later point of differentiation (day 25) in MSCA-1^+^ versus MSCA-1^−^ cells.

In summary, in comparison to cells separated by EasySep, MACS isolated fractions reveal a higher purity and recovery of MSCA-1^+^ cells however, being less viable but retaining their biological function after the magnetic separation. Probably due to the reduced viability, a retardation of the JPC mineralization process was observed using the MACS technology. Nevertheless, we recommend the MACS technology for increasing the purities and recoveries of rare MSCA-1^+^ cells from the entire jaw periosteum tissue.
